# Transcriptomic Analyses Revealed Systemic Alterations in Gene Expression in Circulation and Tumor Microenvironment of Colorectal Cancer Patients

**DOI:** 10.3390/cancers11121994

**Published:** 2019-12-11

**Authors:** Hibah Shaath, Salman Toor, Varun Sasidharan Nair, Eyad Elkord, Nehad M. Alajez

**Affiliations:** 1 College of Health & Life Sciences, Hamad Bin Khalifa University (HBKU), Qatar Foundation (QF), Doha PO Box 34110, Qatar; hshaath@hbku.edu.qa; 2 Cancer Research Center, Qatar Biomedical Research Institute (QBRI), Hamad Bin Khalifa University (HBKU), Qatar Foundation (QF), Doha PO Box 34110, Qatar; mstoor@hbku.edu.qa (S.T.); vsnair@hbku.edu.qa (V.S.N.)

**Keywords:** colorectal cancer (CRC), peripheral blood mononuclear cell (PBMC), transcriptome sequencing, differential expression, immune regulation, inflammation, disease biomarkers

## Abstract

Colorectal cancer (CRC) is among the leading causes of cancer-related deaths worldwide, underscoring a need for better understanding of the disease and development of novel diagnostic biomarkers and therapeutic interventions. Herein, we performed transcriptome analyses on peripheral blood mononuclear cells (PBMCs), CRC tumor tissue and adjacent normal tissue from 10 CRC patients and PBMCs from 15 healthy controls. Up regulated transcripts from CRC PBMCs were associated with functions related to immune cell trafficking and cellular movement, while downregulated transcripts were enriched in cellular processes related to cell death. Most affected signaling networks were those involved in tumor necrosis factor (TNF) and interleukin signaling. The expression of selected immune-related genes from the RNA-Seq data were further validated using qRT-PCR. Transcriptome analysis of CRC tumors and ingenuity pathway analysis revealed enrichment in several functional categories related to cellular movement, cell growth and proliferation, DNA replication, recombination and repair, while functional categories related to cell death were suppressed. Upstream regulator analysis revealed activation of ERBB2 and FOXM1 networks. Interestingly, there were 18 common upregulated and 36 common downregulated genes when comparing PBMCs and tumor tissue, suggesting transcriptomic changes in the tumor microenvironment could be reflected, in part, in the periphery with potential utilization as disease biomarkers.

## 1. Introduction

Colorectal cancer (CRC) is among the most common malignancies in both men and women worldwide. Recent statistics showed that 1 in 22 men and 1 in 24 women face the risk of developing colorectal cancer in their lifetime [1]. Despite extensive research into causes and therapies, CRC remains the second leading cause of cancer deaths, with 51,020 deaths expected during 2019 in the United States [2]. This highlights the need to develop novel biomarkers for early detection and more efficacious therapeutic interventions. Currently, the most common interventions include a combination of surgery, chemotherapy and radiotherapy [3].

As with most common cancers, the effects of environmental factors advocate for better lifestyle choices, including diet and exercise. In addition to sporadic cases of colorectal cancer, a significantly higher occurrence has been observed with evidence of family history of the disease, presented at earlier stages of life [4]. Up to 20% of cases can be attributed to inherited mutations and these fall into two main groups; familial adenomatous polyposis (FAP) related to mutations in the *APC* gene and hereditary nonpolyposis colon cancer (HNPCC)—also known as Lynch syndrome—attributed to mutations in several DNA mismatch repair genes such as *MSH2, MSH6*, *MLH1* and *PMS2* [5,6]. Colorectal cancer also presents high tumor recurrence of up to 50% [7]. Evidence showed the presence of cancer stem cells with the ability to self-renew and re-form tumors in the colon with added drug resistance [8]. Additionally, CRC exhibits high metastasis rates, where most CRC tumors are diagnosed through detection of a secondary lesions in the liver or lungs, rather than the primary tumor in the colon itself [9]. The ability of CRC cells to metastasize alludes to a high rate of epithelial-mesenchymal transition (EMT). This process begins with malignant cells losing their epithelial characteristics including cell–cell adhesions through integrins, which allows for increased mobility and the potential to metastasize to other organs [10].

In perusal of alternatives strategies for disease identification, monitoring and therapy, great efforts have been made for the implementation of a non-invasive tool for CRC characterization. Such strategies are likely to widen acceptance among patients and increase their willingness to participate, leading to potential earlier detection and faster diagnosis. Current tools such as colonoscopies are costly and uncomfortable for patients. Biomarker discoveries will aid in reducing the need for conventional approaches and enable mass screening through peripheral blood analysis. Ciarloni et al. previously reported the characterization of a 29-gene panel in peripheral blood mononuclear cells (PBMCs) for the detection of colorectal adenomas and carcinomas using a nanoliter high throughput qPCR platform for the development of a novel minimally-invasive test [11].

Our knowledge on the link between cancer and immune surveillance has expanded substantially in recent years. Manipulating the immune system, thus making it more hostile towards tumors, presents an inviting target for extensive research. With the rising success rates of immunotherapy on cancers such as melanoma and non-small-cell lung carcinoma (NSCLC), clinical trials on other solid tumor such as CRC suggest a combination therapy approach to tackling tumors that have undergone immune evasion. Food and Drug Administration (FDA) approved CTLA4 inhibitors such as ipilimumab, PD1 inhibitor nivolumab and PD-L1 inhibitor pembrolizumab in high mismatch repair-deficient high metastatic CRC [12], along with newly emerging drugs in combination with or without radiotherapy are just some of the ongoing clinical trials on CRC patients. Single cell transcriptome analysis of tumor infiltrating T cells (TILs) gave rise to the identification of 20 different T cell subsets, each with distinct functions, associations and clonalities, highlighting the complex and dynamic relationship between T cell function and cancer [13]. These data instigated the development of a web tool enabling TIL characterization using the database on a single-cell level, potentially furthering our understanding of immune cell functions in CRC [14].

Recent findings in transcriptome analyses have given us a greater understanding of the mechanisms behind the onset and progression of CRC. We have previously shown that CRC patients have significantly higher levels of immature and granulocytic myeloid cells in the tumor microenvironment and granulocytic myeloid cells in circulation, which were associated with advanced stages and poorly differentiated tumors [15]. However, despite the changes in the composition of immune cells in the tumor microenvironment, whether these changes are reflected in the circulation of CRC patients using transcriptomic approaches is not fully addressed.

In the current study, we undertook a comprehensive approach to compare the transcriptome of PBMCs derived from CRC patients and healthy individuals to the transcriptome of CRC tumor and adjacent normal tissue. Our data revealed similarities and differences in the transcriptome from PBMCs and tumor tissues, suggesting systemic effects of tumorigenesis on cells of the immune system, with potential utilization as disease biomarkers. Further bioinformatics, downstream effectors and mechanistic networks revealed deeper insight into the signaling and functional networks affected in the circulation and tumor tissue of CRC patients.

## 2. Results

### 2.1. Transcriptome Analysis of PBMCs From CRC Patients Revealed Systemic Changes in Gene Expression 

Whole transcriptome RNA-seq analysis was carried out on PBMCs isolated from 10 CRC patients and 15 healthy individuals. Patients’ characteristics are shown in Table 1. Using 2.0 fold change (FC) and ≤0.05 corrected *p* value, 260 transcripts were upregulated and 508 were downregulated (Table S1).

**Figure 1:**
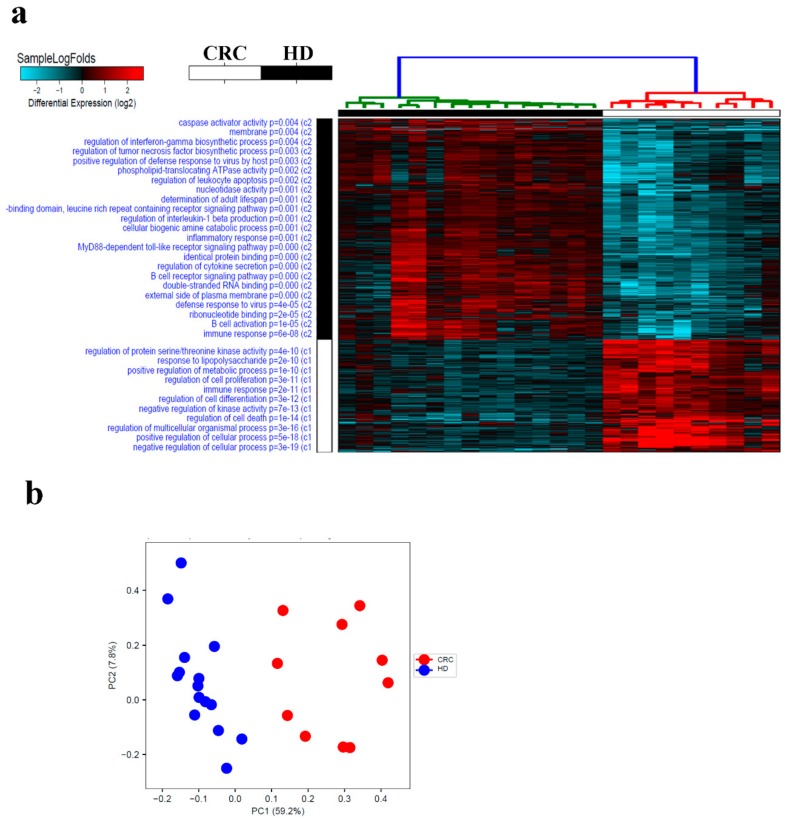
Transcriptional landscape in peripheral blood mononuclear cells (PBMCs), from colorectal cancer (CRC) and health donors (HD). (**a**) Hierarchical clustering of CRC (*n* = 10) and normal (*n* = 15) based on differentially expressed RNA transcripts in PBMCs from each group. Each column represents one sample and each row represents a transcript. Expression level of each transcript (log2) in a single sample is depicted according to the color scale. (**b**) Principal component analysis (PCA) for the RNA transcriptome of PBMCs from CRC and HD.

**Figure 2:**
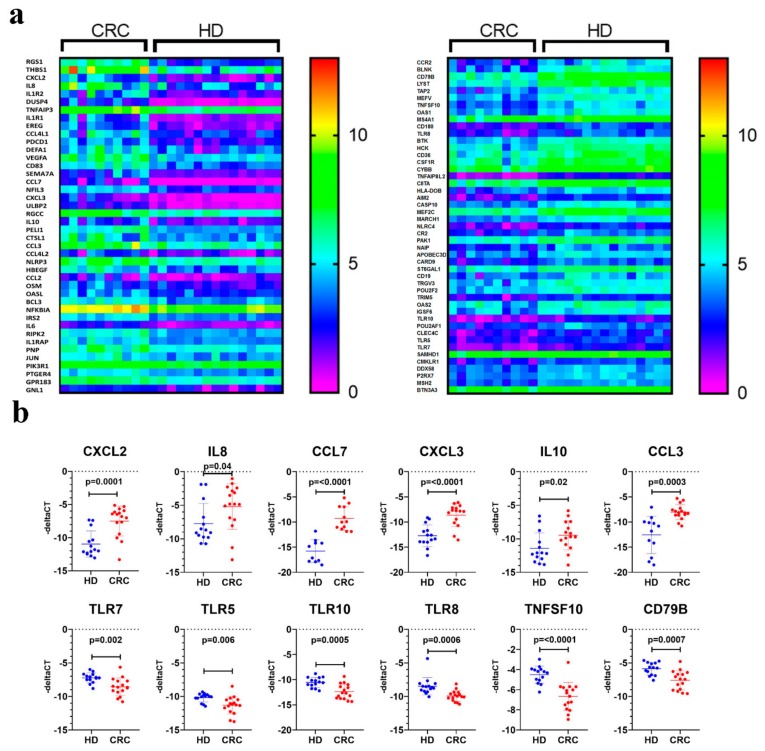
Altered immune signature expression in PBMCS from CRC and healthy donors (HD). (**a**) Heatmap depicting the expression of several upregulated (left) and downregulated (right) immune regulators in PBMCs from ten CRC and 15 healthy individuals. Data are presented as log2 Transcripts Per Million (TPM) expression value. Expression values are depicted according to the color scale. (**b**) The expression of six upregulated (CXCL2, IL8, CCL7, CXCL3, IL10 and CCL3) and six downregulated (TLR7, TLR5, TLR10, TLR8, TNFSF10 and CD79B) based on RNA-Seq data in PBMCs from eight CRC compared to eight health subjects (ran in duplicate) using qRT-PCR. Data are presented as scatter plot (x axis) while –delta CT value is presented on the y-axis.

Sample log fold hierarchical clustering based on differentially expressed transcripts revealed distinct clustering of CRC from healthy subjects (Figure 1a). A number of cellular processes and functional categories were enriched in PBMCs from CRC (regulation of cellular process, regulation of cell death, regulation of kinase activity, regulation of cell differentiation, immune response, regulation of cell proliferation, positive regulation of metabolic process, response to liposaccharide and regulation of protein serine/threonine kinase activity). Enriched genes within each functional category is shown in Table S2. Similar distinction was observed using principle component analysis (PCA; Figure 1b). The list of genes included in PC1 and PC2 are presented as Table S3. Interestingly, distinct immune response clusters were observed in PBMCs from CRC compared to healthy subjects. An immune signature consisting of BCL3, CCL2, CCL3, CCL4L1, CCL4L2, CCL7, CD83, CTSL1, CXCL2, CXCL3, DEFA1, DUSP4, EREG, GNL1, GPR183, HBEGF, IL10, IL1R1, IL1R2, IL1RAP, IL6, IL8, IRS2, JUN, NFIL3, NFKBIA, NLRP3, OASL, OSM, PDCD1, PELI1, PIK3R1, PNP, PTGER4, RGCC, RGS1, RIPK2, SEMA7A, THBS1, TNFAIP3, ULBP2 and VEGFA, was enriched (*p* = 2.6 × 10^−11^) in PBMCs from CRC. On the other hand, an immune signature consisting of AIM2, APOBEC3D, BLNK, BTK, BTN3A3 CARD9, CASP10, CCR2, CD180, CD19, CD36, CD79B, CIITA, CLEC4C, CMKLR1, CR2, CSF1R, CYBB, DDX58, HCK, HLA-DOB, IGSF6, LYST, MARCH1, MEF2C, MEFV, MS4A1, MSH2, NAIP, NLRC4, OAS1, OAS2, P2RX7, PAK1, POU2AF1, POU2F2, SAMHD1, ST6GAL1, TAP2, TLR10, TLR5, TLR7, TLR8, TNFAIP8L2, TNFSF10, TRGV3 and TRIM5 was enriched (*p* = 6.7 × 10^−8^) in PBMCs from healthy individuals (Figure 2a).

Looking further into the altered expression patterns of certain immune related transcripts, the expression of six up-regulated (CXCL2, IL8, CCL7, CXCL3, IL10 and CCL3) and six down-regulated (TLR7, TLR5, TLR10, TLR8, TNFSF10 and CD79B) transcripts was validated using qRT-PCR, which was concordant with the RNA-Seq data. (Figure 2b).

### 2.2. Downstream Effector Analysis of Differentially Expressed Genes in PBMCs from CRC and Healthy Subjects

**Figure 3:**
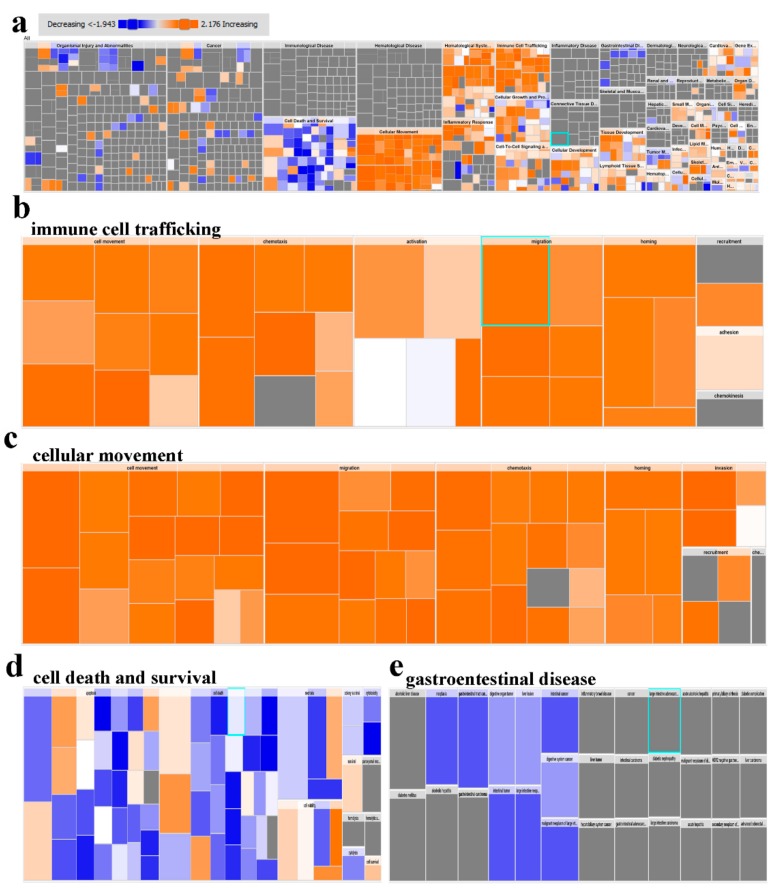
Downstream effector analysis of differentially expressed gene transcripts in PBMCs from CRC and health subjects. (**a**) Tree map (hierarchical heat map) depicting affected functional categories based on differentially expressed genes where the major boxes represent a category of diseases and functions. Illustration of the Immune cell trafficking (**b**), cellular movement (**c**) and cell death and survival (**d**) is depicted. Each individualy colored rectangle is a particular biological function or disease and the color range indicates its predicted activation state—increasing (orange) or decreasing (blue). Darker colors indicate higher absolute Z-scores. In this default view, the size of the rectangles is correlated with increasing overlap significance.

Further analysis, such as downstream effector analysis, provides deeper insight into the contribution of those genes into cellular and functional processes. Ingenuity pathway analysis (IPA) disease and function analysis provides global view of altered functional categories in relation to changes in gene expression. Figure 3a illustrates a high level tree map, exhibiting affected functional categories in PBMCs from CRC compared to healthy individuals based on RNA-Seq data. Remarkably, enrichment in functional categories related to immune cell trafficking and cellular movement was observed in PBMCs from CRC patients (Figure 3b,c). On the other hand, functional categories related to cell death and gastrointestinal disease were under presented in PBMCs from CRC (Figure 3d,e).

### 2.3. Mechanistic Network Analysis Predicts Multiple Altered Signaling Networks in PBMCs from CRC Compared to Healthy Subjects

**Figure 4:**
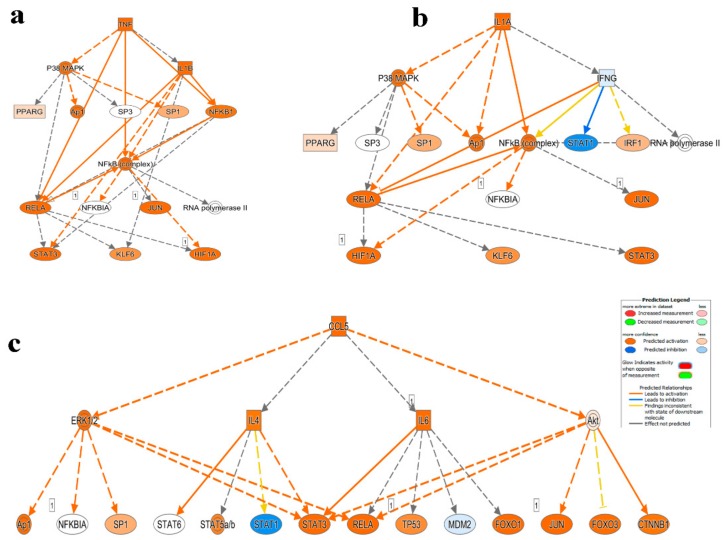
Mechanistic network analysis predicts multiple altered signaling networks in PBMCs from CRC compared to health subjects. (**a**) Illustration of the TNF, (**b**) IL1A and (**c**) CCL5 mechanistic networks. Figure legend illustrate the relationship between molecules within the network.

Mechanistic network analysis is a powerful tool in predicting how alterations in the transcriptome observed in CRC patients, in comparison to healthy donors, could have a downstream effect on signaling networks. Network analysis on the differentially expressed genes using IPA revealed enrichment in several mechanistic networks (i.e., TNF, IL1A and CCL5) in PBMCs from CRC patients (Table S4). Figure 4a highlights the predicted activation state of TNF and its direct activational effects on several downstream molecules with strong confidence (IL1B, NFkB, RELA, NFKB1 and P38 MAPK), these molecules in turn, have further downstream activational effects on genes such as STAT3, HIF1A and JUN, all with high confidence. This highlights the cascading effect of the activational state of TNF and its ability to influence other genes in a complex network, with high confidence, both directly and indirectly.

IL1A, as shown in Figure 4b, has an activating effect on P38 MAPK, Ap1, RELA and NFkB complex predicted with high confidence and IFNG with low confidence. Each of these downstream molecules affects some of the same reoccurring genes including PPARG, JUN, SP1, NFkB, HIF1A, KLF6 and STAT3. IFNG, through IL1A, is predicted to activate IRF1 interferon regulator with less confidence and to inhibit (inconsistence findings) STAT1. STAT1, STAT3, RELA, Ap1 and SP1 are also activated or in the case of STAT1, inhibited by CCL5 indirectly through the effects of IL4, ERK1/2 and Akt (Figure 4c).

IPA predictions gave rise to the enrichment of multiple cancer-associated networks. These networks are comprised of several regulators including enzymes, growth factors, kinases, transporters and so forth. The color intensities of the symbol alludes to the degree of activation or suppression of the respective network component. Figure S1a illustrates TP53 (tumor suppressor) at the center of the top predicted network (cancer, organismal injury and abnormalities and reproductive system disease) directly and indirectly activating 13 downstream effectors and suppressing 15 with varying intensities. The NFkB complex’s effect on cellular functionality, through IPA predictions is also illustrated (Figure S1b) with varying activation (red) and suppression (green) effects on molecules involved in cell to cell signaling, cellular movement and hematological system development and function.

### 2.4. Alterations in CRC Tumor Tissue Compared to Adjacent Normal Tissue 

**Figure 5:**
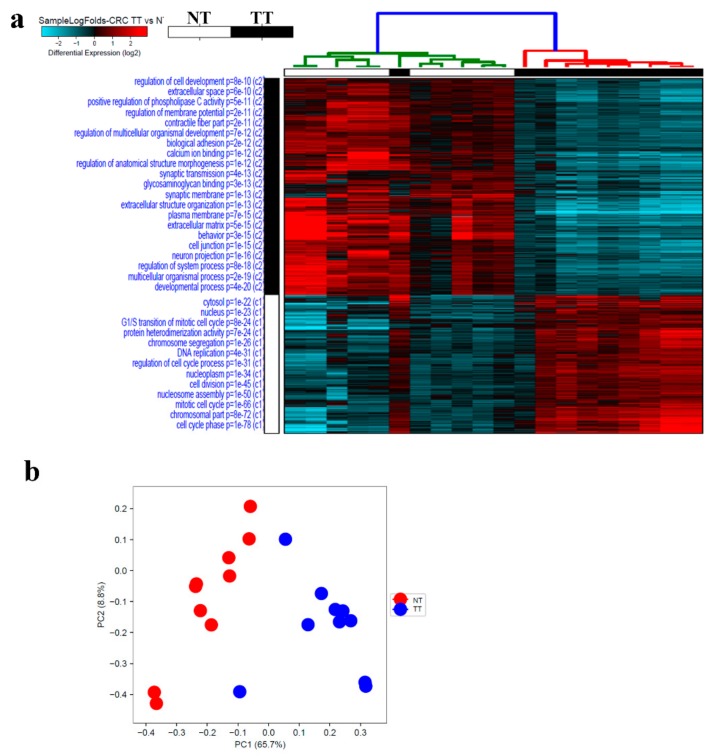
Transcriptional landscape in CRC compared to adjacent normal tissue. (**a**) Hierarchical clustering of CRC (*n* = 10) and adjacent normal tissue (*n* = 10) based on differentially expressed RNA transcripts between the two group. Each column represents one sample and each row represents a transcript. Expression level of each transcript (log2) in a single sample is depicted according to the color scale. (**b**) Principal component analysis (PCA) for the RNA transcriptome of CRC and adjacent normal tissue.

To gain more insight into changes in gene expression in CRC, RNA-Seq and transcriptome analysis were conducted on the same cohort of 10 CRC and adjacent normal tissue which revealed distinct clustering of CRC and adjacent normal tissue based on differentially expressed transcripts presented as sample log fold hierarchical clustering (Figure 5, Table S5). Using 2.0 fold change (FC) and ≤0.05 corrected *p* value, 634 transcripts were upregulated and 991 were downregulated. PCA summarizing the dimensionality of the data set also showed clear distinction between CRC tissue and adjacent normal tissue, with the exception of one tumor sample which clustered with the adjacent normal group (Figure 5b). The differentially expressed transcript identified from current study were compared to differentially expressed genes in colon adenocarcinoma (COAD) from the TCGA/GTEx datasets, which revealed large degree of similarity (Figure S2). A distinct pattern of enriched cellular processes was observed in the tumor tissue (TT) versus normal tissue (NT). Many of the enriched cellular processes in CRC were those involved in cell cycle processes such as DNA replication, chromosome segregation, nucleosome assembly, protein heterodimerization activity and cell division. Suppressed functional categories in CRC compared to NT include biological adhesion, calcium ion binding, synaptic transmission and regulation of cell development (Figure 5a).

### 2.5. Multiple Altered Functional Categories and Signaling Networks in CRC

**Figure 6:**
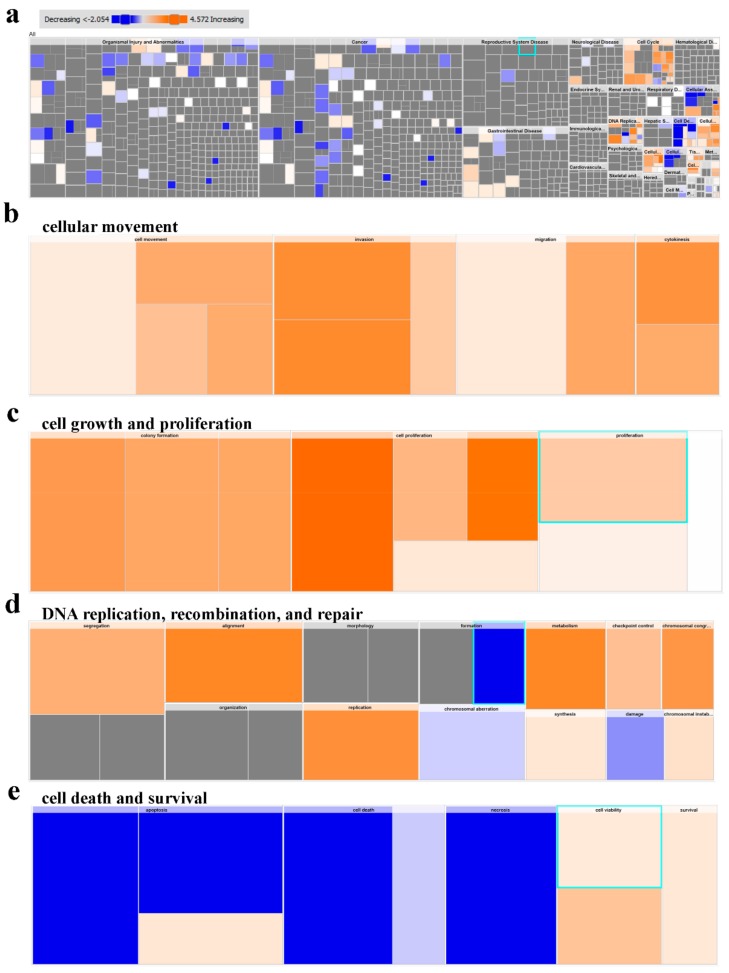
Downstream effector analysis of differentially expressed gene transcripts in Tumor Tissue and adjacent normal tissue. (**a**) Tree map (hierarchical heat map) depicting affected functional categories based on differentially expressed genes where the major boxes represent a category of diseases and functions. Illustration of cellular movement (**b**), cell growth and proliferation (**c**), DNA replication, recombination and repair (**d**) and cell death and survival (**e**) is depicted. Each individual colored rectangle is a particular biological function or disease and the color range indicates its predicted activation state—increasing (orange) or decreasing (blue). Darker colors indicate higher absolute Z-scores. In this default view, the size of the rectangles is correlated with increasing overlap significance.

**Figure 7:**
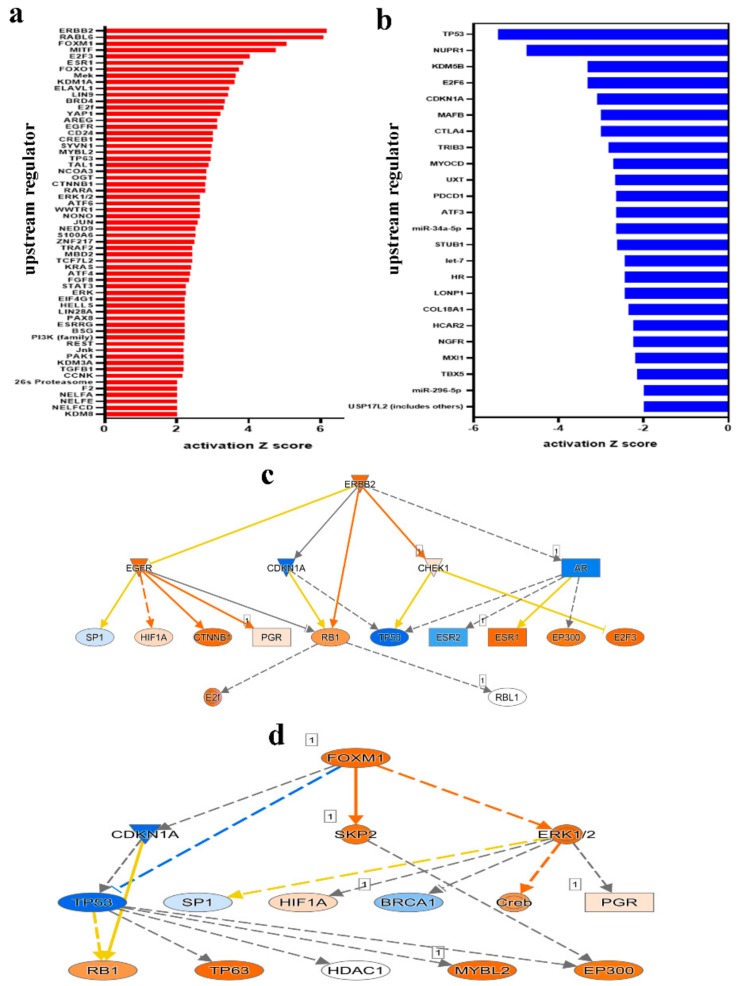
Multiple altered functional categories and signaling networks in CRC. Bar chart depicting activated (**a**) and inhibited (**b**) upstream regulators networks in CRC compared to adjacent normal tissue. X axis represent the activation Z score. Illustration of the ERBB2 (**c**) and FOXM1 (**d**) networks is shown. Figure legend illustrate the relationship between molecules within the network.

IPA conducted on differentially expressed transcripts reveals several activated networks in CRC tissue. Figure 6a shows a high level heat map highlighting altered functional categories in CRC tissue compared to adjacent normal tissue based on RNA-Seq data. Functional categories includes those involved in cellular movement, growth and proliferation, DNA replication, recombination and repair were activated (Figure 6b–d), while the under presented functional category in tumor tissue were mostly those involved in cell death (Figure 6e). Further upstream regulator analysis, presented as bar chart, showed a number of activated (Figure 7a) and inhibited (Figure 7b) networks. Among the top enriched networks, ERBB2 was a common upstream regulator for several differentially expressed genes in our cohort (Figure 7c). Our data findings predict the inhibition of Androgen receptor (AR) via ERBB2 with low confidence, as well as CDKN1A, in turn, inhibiting TP53 and ESR2 respectively and activating RB1 (inconsistent), E2f, ESR1 and EP300. ERBB2 is also shown to activate CHEK1 and EGFR (inconsistent), which activate HIF1A, CTNNB1 and RB1, while inhibiting SP1. The FOXM1 network was also highly enriched in CRC tissue (Figure 7d), which in turn activates ERK1/2 and SKP2 as predicted from our data. As seen in the ERBB2 pathway, CDKN1A is again inhibited via FOXM1, leading to TP53 inhibition both directly and indirectly. Many of the same downstream genes are shown to be affected in both pathways, highlighting the intricacy and complexity of the cascading effects.

### 2.6. Commonality and Differences in Gene Expression Between PBMCs and CRC Tumor Tissue 

**Figure 8:**
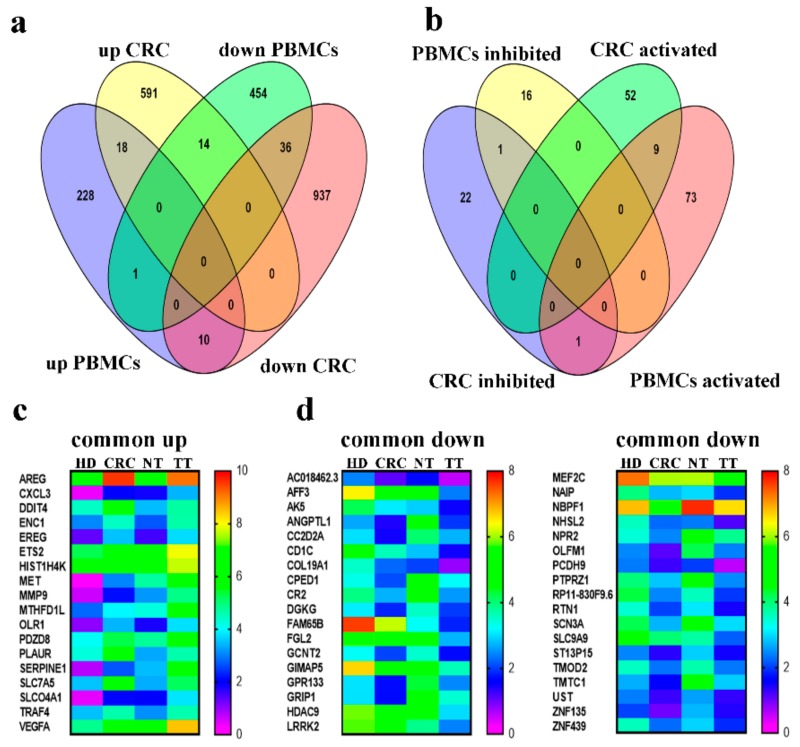
Commonality and differences in gene expression between PBMCs and CRC tumor tissue. Venn diagram showing common differentially expressed transcripts comparing CRC and PBMC data. Upregulated in CRC compared to adjacent normal tissue, downregulated in CRC compared to adjacent normal tissue, upregulated in PBMCs from CRC patients compared to healthy individuals and downregulated in PBMCs from CRC patients compared to healthy individuals (**a**). Commonly inhibited and activated upstream regulators in CRC versus PBMC (**b**). Heat map showing the eighteen common up (**c**) and thirty-six common down (**d**) regulated transcripts identified from panel a.

To integrate the findings from this comprehensive study looking at the global changes in both circulating PBMCs and tumor tissue from CRC patients, common up and down regulated transcripts were identified (Figure 8a), along with common up and down regulated upstream effector networks (Figure 8b) as presented by Venn diagrams. Interestingly, 18 common genes were upregulated in both CRC tissue and PBMCs. Figure 8c depicts each of these transcripts and their heat maps in more detail including data from healthy donor PMBCs, CRC PBMCs, normal tissue and tumor tissue. Thirty six common downregulated transcripts between PBMCs and their matched CRC tumor tissues were detected (Figure 8d). Several upstream effectors were also commonly activated in both PBMC and TT (Figure 8b), these include FOXO1, BRD4 ERK1/2, JUN, NEDD9, STAT3, ERK, Jnk, TGFB1, with only one upstream effector inhibited found to be in common (COL18A1). These differentially expressed transcripts in both circulating PBMCs and in tissue can give valuable starting points to decipher the systemic alterations in CRC.

## 3. Discussion

This study contributes to the growing database of transcriptome data related to CRC, in an effort to provide data pertaining to the Middle-East region, where instances of CRC are on the rise, with main emphasis on alteration in the transcriptome in the circulation as well as in the tumor itself [16,17]. In the current study we explored the transcriptome of PBMCs isolated from CRC patients compared to healthy controls. Our data revealed substantial differences in gene expression between the two groups where PBMCs from CRC patients were more enriched in functional categories related to response to regulation of cell death, kinase activity, cell differentiation, immune response, cell proliferation, positive regulation of metabolic process, response to liposaccharide and regulation of protein serine/threonine kinase activity. On the other hand, PBMCs from healthy subjects exhibited different immune signature indicative of B cell activation, regulation of cytokine and response to viruses. Our data highlights a clear shift in the transcriptome of PBMCs from cancer patients, suggesting such alterations are not restricted to the tumor microenvironment but rather are reflected systematically. Our data is in agreement with previous reports also documenting changes in gene expression in peripheral blood and/or tumor tissue from patients with CRC using digital gene expression-tag profiling approach [18], high through-put real time polymerase chain reaction (PCR) [11], single-cell sequencing [19], human transcriptome array (HTA) [20] and whole genome sequencing [21,22].

IPA downstream effector analyses revealed enrichment in functional categories related to cellular movement, immune cell infiltration, while processes related to cell death were diminished. Mechanistic network analysis revealed activation of multiple networks related to TNF, IL1A and CCL5 signaling. Interestingly, several chemokines such as IL-8 and CXCL3, which promote chemotaxis, migration and angiogenesis and IL-10 and CXCL2, responsible for immunoregulation and inflammation, were shown to be significantly up-regulated in the CRC cohort. Other up-regulated transcripts include cytokines CCL3 and CCL7, with a notably significant up-regulation of expression in CRC PBMCs, indicating higher levels of macrophage function and leukocyte recruitment. As for transcripts that were down-regulated in PBMCs from CRC patients, several of these were in the toll-like receptor (TLR) family. Understandably, the subsided expression of these transcripts could account for the lacking in innate immune response in colorectal cancer. TNFSF10, otherwise known as TRAIL, was also shown to be down-regulated in CRC, reducing the induction of apoptosis in PBMCs CRC patients. qRT-PCR validated the upregulated expression of selected number of genes involved in inflammation and migration, while transcripts involved in innate immune response and apoptosis were significantly down-regulated.

Previous reports identified an upregulation in the expression of CXCL2 and CXCL3, promoting cancer stem cell like properties and tumorigenesis in LoVo colon cancer cells in culture [23,24]. These chemokines, along with IL8 and IL10, also observed to be overexpressed in this study, all play roles in the inflammatory microenvironment of tumors and can even be detected in pre-malignant polyps [25]. Sasaki et al. observed a significant reduction in tumor formation in CCL3 deficient mice compared to wildtype. Studies show that along with CCL5, CCL3 mediated fibroblast accumulation in the tumor microenvironment [26]. Cancer-associated fibroblasts (CAFs) facilitate tumor progression and metastasis through the production of growth factors (such as IL8) and enhancing angiogenesis [27]. CCL7, in concordance with our findings, was also reported to be highly expressed in liver metastasis of colorectal cancer when compared to non-metastatic CRC in 30 patients [28].

Our study shows the downregulation of several TLRs in CRC patients compared to normal controls. Integral to immune cell response, TLRs provide the first point of recognition of foreign bodies or pathogen-associated molecular patterns (PAMPs) once they have evaded physical barriers such as the skin or gut [29,30]. The Toll/IL-1 receptor (TIR) domain of TLRs, found in the majority of TLRs including the ones downregulated in our CRC cohort, in association with myeloid differentiation primary-response protein 88 (MyD88), is responsible for downstream signaling including NF-kB activation and inflammatory cytokine induction to illicit an adaptive immune response [31]. TLR7, shown to be down regulated in our study, has been proposed in the use of its ligand as a potential immunotherapeutic reagent [32]. Loxoribine, a TLR7 ligand was shown to inhibit tumor growth in colon and lung cancer xenografts in vivo. Wang et al. proposed a mechanism of reversing Treg mediated suppression via Loxoribine activation of TLR7, promoting CD4^+^ T cell proliferation and activation [32]. This is complementary to our findings showing a down regulation of TRL7 in CRC patients. With less TLR7 expression in PBMCs of those affected, this depletes the chances of TRL7 antagonist mediated Treg suppression, aiding in the progression of tumorigenesis. Loxoribine has also been shown to inhibit the metastasis of B16 melanoma cells, with significantly better results when given in combination with IL2 injections [33]. Furthermore, TLR7 agonists such as FDA-approved imiquimod has been used in the treatment of superficial basal cell carcinomas (sBCCs) and dermatological malignancies [34,35], with complete tumor clearance after just 6 weeks of treatment with no recurrence [35]. This, yet again highlights the important role of the NF-κB signaling pathway in CRC as we have shown and as reported in previous studies [36,37]. In addition to this, polymorphism in the *TLR7* gene have been suggested to predict the outcome of patients with metastatic colorectal cancer in response to chemotherapy. Longer progression free survival was recorded for patients with *TLR7* rs3853839 G/G variant (11.6 months) in comparison to those with a C variant at the same locus (9.1 months)[38]. Studies on TLR5 have shown similar results to data found on other members of the TLR family. TLR5 activation via flagellin significantly reduced CRC tumor xenografts in size indicating high immune response which could be utilized as potential immunotherapy [39]. Polymorphisms in *TLR5* (rs5744174/F616L) have also been associated with increased survival through their effects in reducing cytokine IL-6 levels [40], which has been linked with a disadvantageous prognosis through IL-6 interaction with STAT3 in CRC [41]. In a study by Grimm et al. TLR7, -8, -9 and -10 expression was significantly upregulated in CRC tumor tissue but interestingly rarely detected in TILs [42]. Several other studies have associated high TLR expression levels with tumor progression [43,44,45]. However, our data revealed suppression of TLR5, TLR7, TLR8 and TLR10 in PBMCs from CRC patients, suggesting a possible flip in the expression of those receptors between the tumor and periphery which warrants further investigation.

To gain further insight into finding a potential gene signature associated with CRC, tumor tissue from the same cohort and their adjacent normal tissue were subjected to whole transcriptome RNA-seq analysis. Mechanistic network analysis has identified a number of activated and inhibited upstream regulators based on the differentially expressed genes in CRC. Of the most significantly activated are ERBB2, RABL6, FOXM1 and MITF. The overexpression of ERBB2, also commonly known as human epidermal growth factor receptor 2 (HER2), is widely associated with approximately 15–30% of breast cancers (HER2-positive) [46]. Overexpression of HER2 has also been documented in gastric cancer [47], salivary duct carcinoma [48] and ovarian cancer [49]. In a cetuximab clinical trial on patients with CRC, those with HER2 amplification had shorter progression free survival after treatment. The study suggests that HER2 may confer resistance to cetuximab treatment in patients with wildtype *RAS* and *BRAF* [50]. ERBB2 copy number amplification was also evident in CRC liquid biopsies in patient plasma. Siravegna et al. reported that when 3 or more copies of ERBB2 were found in circulation, this led to reduced progression free survival in 46 patients [51]. Even though we did not observe ERBB2 overexpression at the transcript level, upstream analysis shows it as a common denominator for several of our up-regulated transcripts, which suggest an activated state of ERBB2 in CRC.

FOXM1, a transcription factor upregulated in several solid tumors, plays a key role in cell cycle progression and tumorigenesis [52]. We previously reported FOXM1 to be a novel target for epigenetic regulation by the miR-320 family in CRC [53]. An inverse correlation was found whereby the overexpress of miR-320 inhibited the progression associate characteristics of colon cancer such as colony formation and migration and sensitized cancer cells to DNA damage inducing agents which could translate to prolonged disease free survival in vivo [53]. Furthermore, FOXM1-dependent CRC cell migration and invasion was described by Li et al. whose results show miR-149 could be responsible for suppressive activity, acting as tumor suppressor in CRC [54]. Several other groups have reported over expression of FOXM1 in CRC. One group has suggested the FOXM1 mechanism of action to be related to EMT transition. Downregulation of FOXM1 resulted in a significant increase in E-cadherin and a decrease in N-cadherin expression, promoting tumor cell invasion [55]. The same group describe a potential link between FOXM1 and β-catenin in CRC tissues, as they found that both were upregulated together compared to normal tissue. As a major player in the Wnt signaling pathway, β-catenin plays an important role in regulating cell growth and adhesion, therefore its inhibition could provide a promising target for CRC therapy [56]. In another previous study, we identified the same upstream effectors (ERBB2, RABL6, FOXM1 and MITF) to be the most effected by palbociclib treatment in the MDA-MB-231 breast cancer cells, reducing colony formation, cell migration and viability [57].

Among the top inhibited upstream regulators in our cohort is TP53. TP53 (or P53) is a tumor suppressor that has been widely studied in relation to cancer susceptibility. In fact, p53-deficient mice develop spontaneous tumors by 6 months due to genome instability [58]. The majority of known mutations in p53 are located in the DNA-binding domain with a dominant negative mode of action [59] and in some cases is known to confer drug resistance [60]. Differentially expressed transcripts in our CRC cohort show inhibition of p53 function as a common upstream regulator, promoting cancer progression.

Being able to perform RNA-seq on both circulating immune cells and tissue from the same patients gives this study the leverage of being able to look for commonalities within the differentially expressed transcripts between the two tissue types. Interestingly, a significant number of transcripts were found in common. This overlap conveys the potential utilization of a less invasive blood sample collection in deciphering the characteristics of a specific tumor microenvironment on an individual basis, allowing for personalized medical care. Eighteen common up regulated transcripts were found between CRC tissue and their respective PBMCs. Among these, AREG, is associated with various types of cancers and inflammatory conditions [61]. Proto-oncogene c-MET, VEGFA, CXCL3 and MMP9, which appear to play roles in CRC biology and immune function [62,63,64] are also up regulated. In addition to this, thirty-six common down regulated transcripts were found in common between CRC PBMCs and their respective tumor tissues. PCDH9, a member of the cadherin superfamily, is among the gene transcripts under expressed in our cohort. Loss of cadherin promotes metastasis via the loss of cell–cell adhesions in cancer [65]. CD1c is a T cell surface glycoprotein, structurally related to MHC molecules [66]. Their decreased expression may affect antigen recognition, leading to deficient immune cell function associated with cancer progression. Many of these genes seem to have unknown function in relation to cancer onset and progression but are known to play roles in other abnormalities (e.g., GRIP1 in Fraser Syndrome [67]). Further studies are required to elicit the exact role that each of these play in cancers and the effects of their differential expression in CRC patients.

## 4. Materials and Methods

### 4.1. Ethics and Sample Collection

All patients included in the study were treatment-naïve and were provided with a written informed consent prior to sample collection. The study was performed under ethical approval from Qatar Biomedical Research Institute, Doha, Qatar (Protocol no. 2017-006). The characteristics of patients and healthy donors included in the current study are provided in Table 1.

### 4.2. PBMC Isolation and RNA Extraction

PBMCs were isolated from whole blood using density gradient centrifugation using Ficoll/Histopaque. The PBMC layer was isolated and washed twice with PBS. RNA was extracted from PBMCs of 10 CRC patients and 15 healthy subjects using the RNA/DNA/Protein Purification Plus Kit (Norgen Biotek Corp, Ontario, Canada) as per the manufacturer’s instructions.

### 4.3. Tumor Tissue and RNA Extraction

Tumor tissues (TT) and adjacent normal tissues (NT) taken away from tumor margins, were cut from freshly resected tissues by pathologist. RNA was isolated using the RNA/DNA/Protein Purification Plus Kit (Norgen Biotek Corp, Thorold, Canada) as per the manufacturer’s instructions from TT and adjacent NT. Briefly, frozen tissues were transferred into a mortar containing adequate amount of liquid nitrogen and were grinded thoroughly using a pestle followed by resuspending the tissue in lysis buffer in an Eppendorf tube followed by RNA extraction protocol.

### 4.4. RNA Concentration and Quality Assessment

The concentration and purity of extracted RNA were measured using NanoDrop 2000c (Thermo Scientific, Waltham, MS, USA). RNA quality and quantity were assessed using the Agilent RNA 6000 Nano Kit (Agilent Technologies, Santa Clara, CA, USA) and Agilent 2100 Bioanalyzer (Agilent Technologies) as per the manufacturer’s instructions. Samples were stored at −80 °C.

### 4.5. Library Preparation and RNA sequencing

The RNA was quantified using Qubit instrument (Invitrogen, Carlsbad, CA, USA) and RNA BR assay kit (Invitrogen). 100 ng of RNA was used as input for library preparation using TruSeq RNA Access Library preparation kit (Illumina, San Diego, CA, USA) as per the manufacturer’s instructions. Briefly, the RNA was fragmented into small pieces under high temperature using divalent cations. The RNA fragments were immediately reverse transcribed to first strand cDNA using random hexamers. Following the first strand, second strand was synthesized by incorporating dUTP instead of dTTP. The sequencing adaptors were ligated to the double-stranded cDNA followed by a single “A” nucleotide adenylation at 3’ end of blunt fragments. The final library was created by capturing the coding regions of the transcriptome using sequence-specific probes. The yield of cDNA libraries was quantified using Qubit dsDNA HS assay kit (Invitrogen) and size distribution of the cDNA libraries were determined using the Agilent 2100 Bioanalyzer DNA1000 chip (Agilent Technologies). The clusters were generated on a cBot cluster generation system (Illumina) and sequencing was done on Hiseq 4000 with 300 bp paired-ends.

### 4.6. RNA-Seq and Ingenuity Pathway Analyses

Exported FASTQ files were subsequently pseudo aligned to the human genome and reads were counted using KALLISTO 0.42.1 and Altanalyze v.2.1.3 software as described before [68,69,70]. Hierarchical clustering was performed using cosine for columns and cosine for rows while principal component analysis was perfumed to assess the relatedness of samples. Functional annotation and enrichment analyses were conducted using Ingenuity Pathways Analysis (IPA) software (Ingenuity Systems; www.ingenuity.com/) as we previously described [70]. Differentially expressed genes in colon adenocarcinoma (COAD) from the TCGA/GTEx datasets were retrieved from GEPIA2 (http://gepia2.cancer-pku.cn/#degenes).

### 4.7. Quantitative Reverse Transcription PCR (qRT-PCR)

Five-hundred ng of RNA was reverse transcribed to get cDNA using the High Capacity cDNA Reverse Transcription kit (Applied Biosystems, Foster City, CA, USA) product code: 4368814 in the Veriti™ 96-Well Thermal Cycler (Applied Biosystems). Up regulation and down regulation of transcripts were validated using qRT-PCR on the QuantStudio 7/6 Flex qPCR (Applied Biosystems). Real time PCR was performed using PowerUp™ SYBR™ Green Master Mix (Applied Biosystems) and relative levels of transcripts were determined from their respective CT values normalized against β-actin transcript levels. Sequences of primers used in the current study are listed in Table 2. The primers were designed using Primer3. (http://www.ncbi.nlm.nih.gov/tools/primer-blast/)

### 4.8. Statistical Analyses

Statistical analyses and graphing were performed using Microsoft excel 2016 and GraphPad Prism 8.0 software (GraphPad, San Diego, CA, USA). The Benjamini–Hochberg False Discovery Rate (FDR) method within AltAnalyze was used to calculate adjusted p values. For comparative qRT-PCR analysis, *p*-values ≤ 0.05 (two-tailed t-test) were considered significant. For IPA analyses, a Z score (2.0 ≤ Z ≥ 2.0) was considered significant.

## 5. Conclusions

In the present study using transcriptome analysis, we identified a number of differentially expressed transcripts reflecting systemic effects of tumorigenesis immune cells, with potential utilization as disease biomarkers. Interestingly, PBMCs from CRC patients exhibited induction of inflammatory immune response. Potential utilization of changes in circulating immune cells as indicator of CRC occurrence and to monitor disease progression and response to therapy warrants further investigation.

## Figures and Tables

**Table 1:** Characteristic Features of Study Populations.

	HD	CRC Patients
**Number**	15	10
**Age** (Median)	30 (21–47) *	41 (36–62) *
**Gender** (Male, Female)	7:8	6:4
**TNM stage**		
I		0
II		3
III		7
IV		0
**Histological grade**		7
Poorly differentiated		
Well/Moderately differentiated		3

HD; Healthy donors.; CRC; Colorectal cancer; *Median range.

**Table 2:** Primer Sequences Used For qRT-PCR With PowerUp™ SYBR™ Green Master Mix.

Gene	Forward Primer (5′-3′)	Reverse Primer (5′-3′)	Product Size
**CXCL2**	GAAAGCTTGTCTCAACCCCG	TGGTCAGTTGGATTTGCCATTTT	82
**IL8**	GGAGAAGTTTTTGAAGAGGGCTG	TTTGCTTGAAGTTTCACTGGCA	95
**CCL7**	TTGCTCAGCCAGTTGGGATTA	GCTCTCCAGCCTCTGCTTAG	86
**CXCL3**	AGATACTGAACAAGGGGAGCAC	TCCTTTCCAGCTGTCCCTAGA	150
**IL10**	TACGGCGCTGTCATCGATTT	TAGAGTCGCCACCCTGATGT	191
**CCL3**	GCTCTCTGCAACCAGTTCTCT	TCGCTTGGTTAGGAAGATGACA	159
**TLR7**	TCAAGAAAGTTGATGCTATTGGGC	ACCATCTAGCCCCAAGGAGT	195
**TLR5**	GTCACCAAACCAGGGATGCT	GGGCAAAGTCAATTGCCAGG	100
**TLR10**	GCCAATTCTGACCGTGTCAAC	CTGCTGAATTCCCACGGCTT	79
**TLR8**	GAAACATGGTTCTCTTGACACTTCA	GGCTTCCTTCAAGGTCTCCT	75
**TNFSF10**	TGAAGCAGATGCAGGACAAGT	TGGTTTCCTCAGAGGTTCTCAAAA	174
**CD79B**	CGGAATCCCAAAGGTAGTGCT	TCCAGAGCCAGCTCACATTG	130

## Supplementary Materials

The following are available online at https://www.mdpi.com/2072-6694/11/12/1994/s1, Figure S1: Enrichment in multiple cancer-associated networks in breast cancer; Figure S2: Common upregulated and common downregulated transcripts identified from current study compared to the TCGA/GTEx COAD dataset; Table S1: List of differentially expressed genes (2.0 fold change and ≤ 0.05 corrected p value) in PBMCs from 10 CRC compared to 15 health individuals; Table S2: Enriched gene in PBMCs from CRC (C1) and PBMCs from healthy donors (C2); Table S3: ENSEMBL IDs for genes used in PC1 and PC2 Principle component analysis; Table S4: Top enriched mechanistic networks in PBMCs from CRC compared to PBMCs from healthy donors; table S5: List of differentially expressed genes (2.0 fold change and ≤ 0.05 corrected p value) in CRC compared to adjacent normal tissue.
